# Lived Experience of Health and Wellbeing Among Young People with Early Psychosis in Aotearoa New Zealand

**DOI:** 10.1007/s10597-024-01259-6

**Published:** 2024-03-16

**Authors:** Victoria Chinn, Ella Creagh, Tracey Gardiner, Briony Drysdale, Pāyal Ramritu, Zara Mansoor, Susanna Every-Palmer, Matthew Jenkins

**Affiliations:** 1 School of Health, Victoria University of Wellington, Te Herenga Waka, Wellington/Te Whanganui-a-Tara, Aotearoa, New Zealand; 2 Department of Psychological Medicine, University of Otago Wellington, Wellington/Te Whanganui-a-Tara, Aotearoa, New Zealand

**Keywords:** Psychosis, Young People, Health, Well-being, Arts-based Methods, Lived Experience

## Abstract

First episode psychosis (FEP) can disrupt a young person’s life and future health. Those with lived experience of FEP can inform effective support. This study investigated how young people with FEP experience good health and wellbeing living in Aotearoa New Zealand. Recent clients of early intervention services (*n* = 12) shared their stories across varying traditional and creative platforms. Thematic analysis revealed seven themes important for living well with FEP: *whanaungatanga* (relationships), *addressing stigma, finding out who I am with psychosis, getting the basics right, collaborative healthcare, understanding psychosis*, and *access to resources.* The themes informed five supporting processes: whakawhanuangatanga (relationship-building), using holistic approaches, creating space for young people, reframing, and improving access to appropriate resources. These findings deepen our understanding of how we can support young people to live well with FEP. This study highlights the value of creative methods and partnering with lived experience experts to conduct meaningful health research.

This trial was registered at Australian New Zealand Clinical Trials Registry (ANZCTR) CTRN12622001323718 on 12/10/2022 “retrospectively registered”; https://www.anzctr.org.au/Trial/Registration/TrialReview.aspx?id=384775&isReview=true.

## Introduction

Young people diagnosed with psychosis face a staggering reduction in life expectancy by up to 20 years (Thornicroft, 2011). This gap, termed the ‘scandal of premature mortality’, arises partly from the concurrent decline in physical health that accompanies psychosis (Thornicroft, 2011). First episode psychosis (FEP) describes the early phase when a person begins to experience symptoms of a psychotic disorder, typically characterised by the onset of distorted and disconcerting sensations and beliefs that deviate from their usual identity. These symptoms can disrupt one’s relationships, occupation, cognitive functioning, and identity, and often lead to social withdrawal (Jordan et al., 2020; Makdisi et al., 2013). Reduced self-efficacy and a lack of motivation can also result from these symptoms, leading to the neglect of everyday health behaviours such as eating well and regular exercise (Gates et al., 2015), and the adoption of risky behaviours to cope with mental distress such as recreational drug use (Mueser & Gingerich, 2013). These changes to health behaviour patterns coupled with the adverse side effects of antipsychotic medication heighten the risk of developing comorbid cardiometabolic diseases (Hultsjö & Blomqvist, 2013), increasing morbidity and premature mortality in this population (Gates et al., 2015). Therefore, early intervention to promote the health and wellbeing of people living with psychosis is critical to reduce these potential adverse health outcomes.

Effective early intervention requires an understanding of what it means to live well with FEP, with recovery playing an important role in this discussion. Specifically, “personal recovery” is considered a more ethical and empowering goal for mental health services compared to “clinical recovery” (Slade, 2009). Slade (2009) describes personal recovery as living a fulfilling life despite one’s condition, and is thus self-defined, informed by lived experience, and can look different for everyone. For some it might mean an absence of symptoms, coming off antipsychotic medication, building holistic health while experiencing ongoing symptoms, or combination of these factors (Pitt et al., 2007). This broadened framing of recovery challenges support services to move beyond symptom treatment (clinical recovery) toward improving everyday life for those living with psychosis (Davidson & Roe, 2007). Despite being an important goal, the flexibility of recovery as a construct leads to confusion over how to define and discuss it. To avoid this confusion, we have chosen to explore health and wellbeing distinct from recovery, while acknowledging these concepts are intertwined.

Similar to personal recovery, health can be conceptualised as living well in the presence or absence of illness (Misselbrook, 2014). Recent research shows that health and wellbeing for people with psychosis is multifaceted and subjective. Lal et al. (2014) found that young adults with FEP view their health as a combination of mental, physical, social, spiritual, financial, and moral factors. Moreover, health support beyond mental wellbeing (e.g., physical health literacy) enables people to live well despite experiencing symptoms (Chee et al., 2019). Wellbeing with psychosis has also been conceptualised as a transition to an enhanced sense of self, signified by relief, hope, empowerment, meaning, self-worth, connection, and ‘good’ feelings (Schrank et al., 2014). Clearly, those seeking to aid young people with psychosis must understand what constitutes health and wellbeing *for them*, rather than solely relying on health service definitions.

Numerous factors can aid or hinder one’s journey to living well with psychosis. Physical health behaviours, social support, cultural values, personal agency, therapeutic healthcare, spirituality, and sharing stories are all identified as important for improving health and wellbeing in this population (Hultsjö & Blomqvist, 2013; Vaingankar et al., 2020). In particular, controllable behaviours in daily life are important for enhancing health and empowerment (Syrén & Hultsjö, 2014), and those that positively contribute to mental health are often prioritised (Hultsjö & Blomqvist, 2013). Not surprisingly, influencing factors are complex, encompassing fundamental needs such as shelter and safety, alongside broader influences like social values and the economy (Schrank et al., 2014). Furthermore, this system of factors is experienced in highly varied ways. A single factor (e.g., services, medication) can possess both harmful and supportive qualities (Cook & Chambers, 2009), further complicating clients’ pathways to improved health. Services thus face the challenge of working with clients beyond diagnosis to identify and address modifiable factors, enhancing support for those with FEP.

Methods centred on lived experience are crucial for understanding how people with FEP experience health and wellbeing and determining effective support. Qualitative methods that position participants as experts are well-suited to investigate their experiences within the complex ecosystem of influencing factors (Fusar-Poli et al., 2022). Honouring such experiences and using them to co-create support systems for health and wellbeing is the basis of strength-based approaches to intervention development such as experience-based co-design (Donetto et al., 2014). Further, methods like interviews and focus groups are useful to obtain rich data that speaks to the complexity of individual stories (Rich & Ginsburg, 1999). However, personal and confusing experiences can be hard to discuss and cannot always be transduced to language (Bagnoli, 2009). Including both visual and verbal methods can be valuable by providing multiple avenues for expression (Attard et al., 2017).

Our research investigated how young people with FEP live well in the context of the Greater Wellington region of Aotearoa New Zealand (Aotearoa hereafter). We sought to answer the following research questions: How do young people with FEP experience good health and wellbeing in Aotearoa? What are the key barriers and facilitators they encounter to improving their health and wellbeing?

## Methods

### Study Design

This study reports on findings from a contextual inquiry workshop that explored young people’s experiences of living well with FEP. This workshop was part of a larger co-design project that aims to develop a support system toward improving their health and wellbeing. Further information about the full co-design project is detailed elsewhere (Jenkins et al., 2023).

The research team comprises researchers at varying career stages with expertise in mental health, physical activity, participatory approaches, programme design, and lived experience of FEP and/or mental distress. Partners within Early Intervention Services in Psychosis (EISP) also informed the project at each stage and assisted with recruitment. EISP is a specialist community mental health team of nurses, psychiatrists, clinical psychologists, social workers, occupational therapists, and Māori health workers who support young people (13–25 years) experiencing psychosis for the first time. Service users are referred to EISP and can receive support up to two years. A Māori health agency, Toi Tangata, supported the project by helping to develop the co-design process to ensure relevance and cultural safety conducting research within Aotearoa alongside the lived experience members of our research team. A group of professional illustrators, ‘The League of Live Illustrators,’ also co-created materials including guiding frameworks and depictions of young people’s perspectives. The research team partnered with EISP clients and the other stakeholders throughout the project to ensure meaningful results for those using services.

Critical to conducting research in Aotearoa is the duty to uphold Te Tiriti o Waitangi (the Māori interpretation of the Treaty of Waitangi), which recognises a shared governance between Māori (the indigenous people of Aotearoa) and the British Crown. In honouring this commitment, the research team partnered with Māori stakeholders toward supporting Māori health. This paper integrates te reo Māori (indigenous language of Aotearoa) to further reflect our commitment to Te Tiriti o Waitangi and uplift mātauranga Māori (Māori ways of knowing) on an international platform. Te reo Māori is taonga (a treasure) and each word typically carries multiple and complex meanings embedding depth to their use. We have attempted to translate these words for an international audience; however, we acknowledge their full meanings cannot be conveyed in English.

This study adopted a phenomenographic approach. Phenomenography explores how people make sense of the world around them (Sjöström & Dahlgren, 2002) and is well-suited to our aims given the subjective and individualised experience of living well with FEP. This approach assumes that people differ in how they experience the world, articulate these differences, and allow others to understand them (Sjöström & Dahlgren, 2002). Building upon previous studies that have used art-based and creative qualitative methods with this population (Attard et al., 2017; Boydell et al., 2012; Lal et al., 2014), we gave participants different ways to engage with the questions including artistic materials. This approach was chosen to reduce barriers to self-expression, supporting participants to share their experiences in a way that felt comfortable to them.

### Recruitment

Rangatahi whai ora^1^ aged 16 years and above with lived experience of FEP were recruited through EISP Wellington. Posters were distributed within EISP and case managers helped identify potential participants. To participate, clients could not be currently experiencing an acute episode of psychosis. An ‘acute episode’ was self-determined as part of the informed consent process. A youth psychologist (Z.M.) also attended the workshop to support if any of the participants felt distressed. Rangatahi whai ora were encouraged to nominate one whānau^2^ member who could provide insights as a key support person after the workshop. Informed consent was obtained prior to participation in the study. Ethics approval was granted by the University of Otago Human Ethics Committee (ref H22/048) and the project underwent Māori consultation in line with guidelines for conducting research in Aotearoa.

### Data Collection

The data were collected during an in-person, four-hour workshop. The first hour was dedicated to welcoming the participants and introducing the project. Everyone was encouraged to ask any questions and reminded they could opt out of the activities or discontinue the workshop at any point. Whakawhanaungatanga^3^ guided the welcome. Everyone was invited to introduce themselves in a way that felt comfortable to them. The introductions were followed by outdoor activities focused on movement and building connections with each other (e.g., a peer-led group yoga session). The remaining three hours included two breakout sessions separated by a lunch break. A quiet room was available for rangatahi whai ora to occupy if needed.

**Fig. 1:**
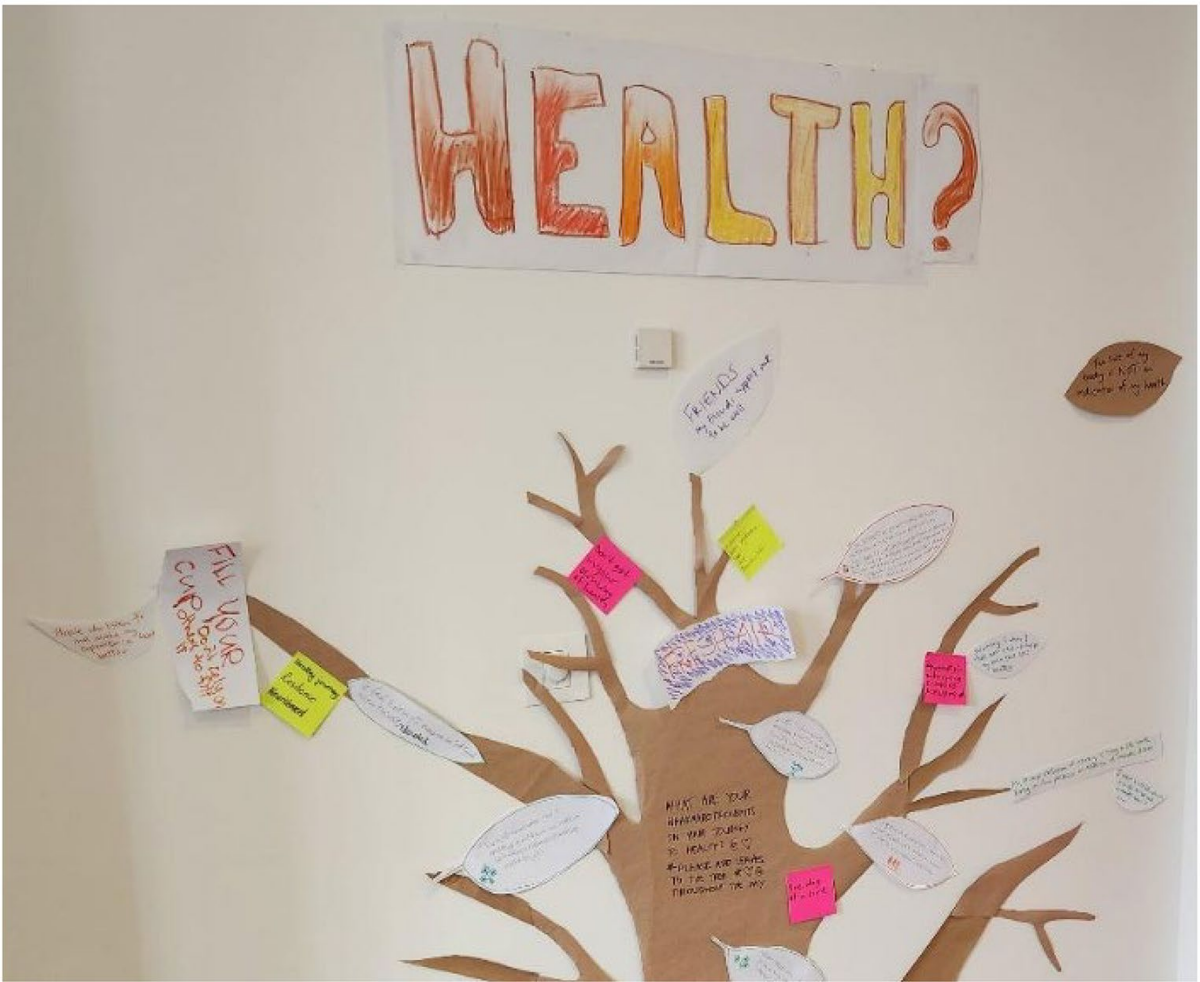
The health tree. Note. Rangatahi whai ora added leaves to the health tree throughout the workshop that contained written responses describing what health and wellbeing meant to them

A purpose-made framework, ‘Navigating the Puna,’ incorporated prompts and questions, which guided rangatahi whai ora to express themselves throughout the workshop (see Jenkins et al., 2023). The first breakout session asked them to share the enablers and barriers they experienced to improving their health and wellbeing (e.g., What helps you feel connected? What needs to change?). The second session encouraged them to explore their vision of wellness and dreams they held for the future (e.g., Where/when have you felt your most well? What’s your dream for yourself?). All questions were presented as a group and participants shared their responses through various platforms including individual or small group discussions, sculptures, poetry, writing, drawings, collage, songs, and Mentimeter (an online platform that captures anonymous, real-time feedback). Participants also added leaves to a tree expressing what health meant to them (see Fig. 1). To aid interpretation of the creative outputs (e.g., sculptures, drawings), they were encouraged to provide descriptions of their works. Each session concluded with a group discussion to further explore their experiences as they felt comfortable.

The workshop was not audio recorded according to participants’ wishes (unless consent was provided for small group discussions). Members of the research team and professional illustrators took notes throughout the workshop capturing observations, meaningful quotes, and group discussion summaries. The research team met immediately after the workshop to compare their notes for analysis. Synergies and discrepancies were discussed to reach consensus on interpretation of the workshop events and resulting works. Participants were also invited to share any follow up ideas or feedback via email for a week after the workshop.

Those unable to attend the workshop were invited to contribute via an online interview. The interviews asked open-ended questions consistent with the workshop outline and ‘Navigating the Puna’ framework. The whānau session followed a similar format, but from the perspective as a key support person.

### Analysis

**Fig. 2:**
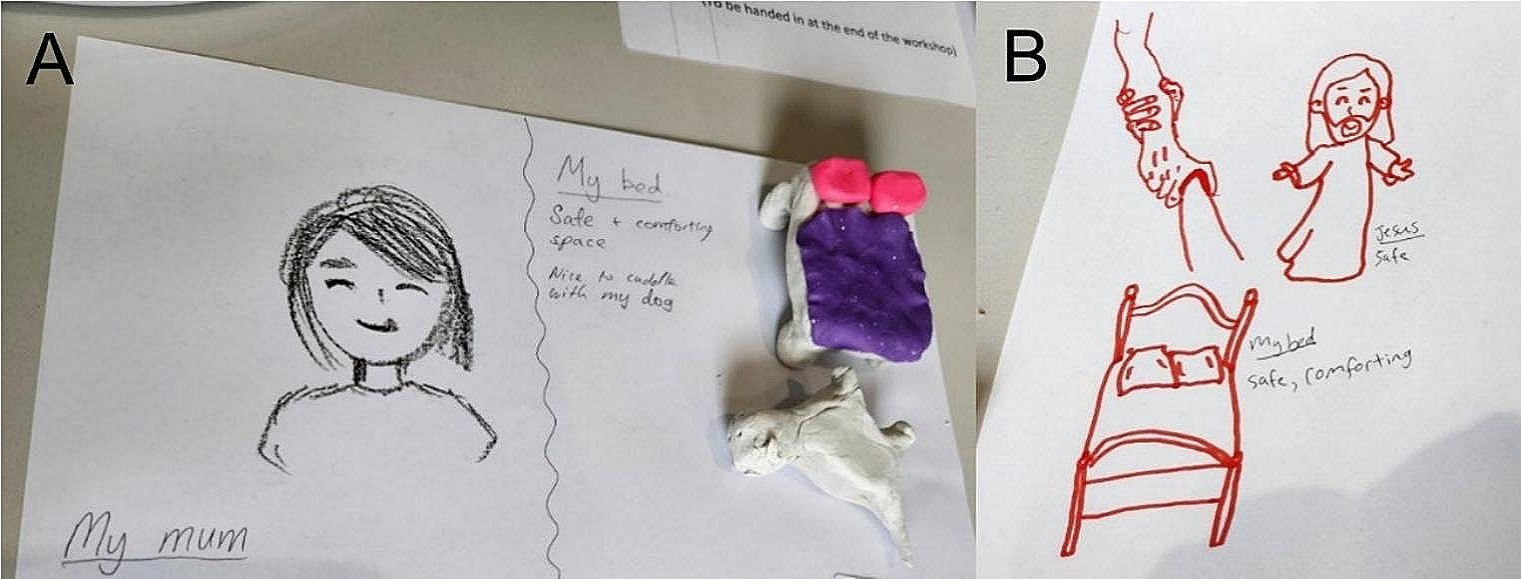
Creative response examples. Note. The images depict creative responses that answer the question, “What factors support your health and wellbeing?” Image A depicts a drawing of their “Mum” who is smiling, indicating someone they see as friendly, warm, and safe. There is also a clay sculpture of their bed described as a “safe and comforting space” and dog with writing “nice to cuddle with my dog”. A focus on the bed and dog may suggest their bed provides a safe space that is comforting through connection with their pet and free of judgement. The colours pink and purple used to sculpt the bed also signify a happy space. Image B is a drawing of Jesus and their bed, both captioned “safe” and “comforting.” The drawing also shows two hands holding each other signifying a connection between people and/or their faith. The image suggests this participant finds safety in their faith, connection, and in the comfort of their bed

We used an expanded hermeneutic phenomenological approach to our analysis, which builds on classic phenomenography by incorporating both written and visual forms of data that are interpreted and analysed together to understand experience (Boden & Eatough, 2014). The workshop data were compiled into a single document. Written responses (e.g., leaves, written entries) were transcribed. Visual data were photographed, and the associated descriptions were transcribed as captions. Similar to Attard et al. (2017), the creative works’ content, colours, and style were considered for interpretation and added to the captions (see Fig. 2 as an example). The field notes were then incorporated into the document to enhance meaning of the dataset by noting the discussion topics, direct quotes, and further annotations that expanded interpretation (Phillippi & Lauderdale, 2017). The interviews were transcribed separately using a web app (otter.ai).

The data were analysed using thematic analysis, a method to identify patterns of meaning in a body of data following several stages: familiarisation with the data, identifying data relevant to the research questions, grouping similar data into categories, and reviewing and refining the categories into themes (Braun & Clarke, 2006). The team reviewed the document and further discussed interpretation across the different types of data and potential themes (familiarisation). The data were then organised by similarity and excerpts from the interviews were added to each category, which deepened and expanded each concept (identifying and grouping relevant data). Two researchers (V.C. and E.C.) conducted the second round of analysis by reviewing, reflecting upon, and refining the categories relative to the research questions, which produced nine preliminary themes. The preliminary themes were further discussed as a team which resulted in the final themes (reviewing and refining).

## Results

Participants were rangatahi whai ora (*n* = 12) who were EISP clients within the past 6 months as well as one whānau member. Eleven rangatahi whai ora and the whānau member attended the workshop. The same whānau member also participated in the follow up whānau session. An additional one-to-one interview took place with a recent EISP client who could not attend on the day. Rangatahi whai ora were aged 19–27 years (*M* = 23 years) and identified as either male (*n* = 6) or female (*n* = 6). Their recorded ethnicities were New Zealand European (*n* = 8), Māori (*n* = 2), Chinese (*n* = 1), and Middle Eastern (*n* = 1).

**Fig. 3:**
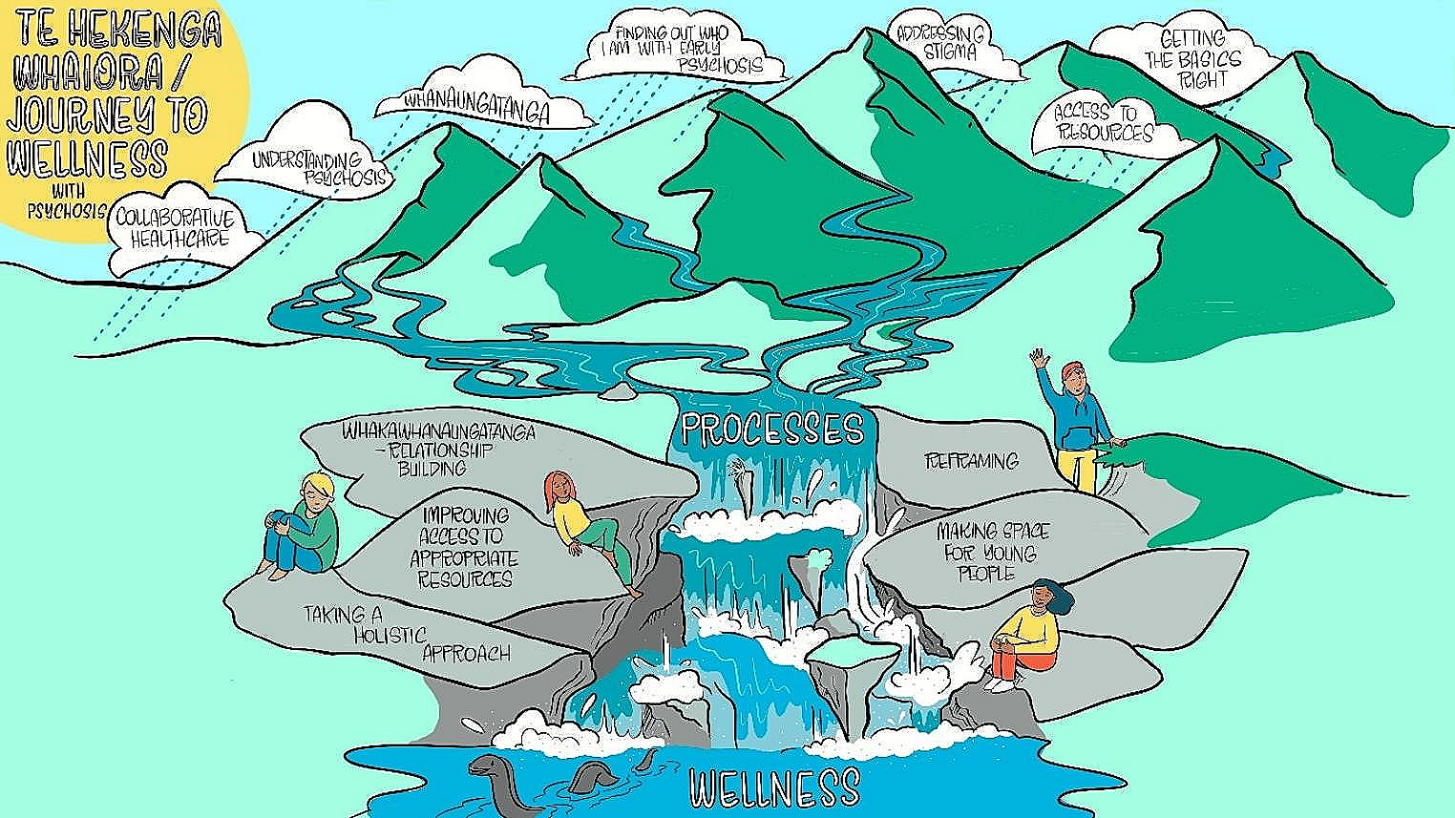
Te Hekenga Whaiora/ The Journey to Wellness framework. Note. The river is a metaphor for the journey of rangatahi whai ora to living well with early psychosis. The clouds depict the seven themes signifying the important health aspects that feed the rivers. The braided rivers symbolise how the seven themes interrelate and interact contributing to rangatahi whai ora feeling well. The five processes that support rangatahi whai ora to improve health and wellbeing are depicted as rocks that aid the river to flow faster as a waterfall into the sea of wellness. While the water pictured here flows in one direction, we acknowledge that feeling well is not necessarily an ‘endpoint’, but rather an ongoing and multi-directional journey

Seven themes emerged that highlighted important aspects for living well with psychosis among rangatahi whai ora: *whanaungatanga*; *addressing stigma*; *finding out who I am with psychosis*; *getting the basics right*; *collaborative healthcare*; *understanding psychosis*; and *access to resources*. While the themes naturally interacted and overlapped, they were distinct concepts. Te Hekenga Whaiora/ The Journey to Wellness framework depicts the seven themes (Fig. 3). All responses originated from rangatahi whai ora unless otherwise noted. The form of expression is also indicated after each response.

### Whanaungatanga

Rangatahi whai ora often felt isolated and detached through early psychosis. Feeling connected and having a social life were described either as being an important part of their wellbeing, or something they strived for. Whanaungatanga is a Māori concept that encompasses connection and relationships, enabling a sense of belonging through kinship and non-kin relationships (e.g., friendship), and more broadly, to ancestors and the natural world (Mead, 2003). Various key supporters were mentioned including partners, friends, family, peers, pets, and others that were there for them, listened, and supported them to be well.

Connections provided support and safety (see Fig. 2). In some cases, rangatahi whai ora turned to their family and pets. Others described feeling closer to, and safer with, friends due to the stigma and/or lack of understanding they experienced from family: “Good friends are important… that’s been my pillar. When I was unwell, that was all I had. My family was there, but not, they just wouldn’t have understood and would have put a lot of stigma on it” (interview). Rangatahi whai ora also found safety in their faith and places that held special meaning and connection: “… [One] of my delusions …while I was unwell was ‘I need to go back to the river.’ That’s my home. It’s really quite powerful” (interview).

Belonging to a group and being accepted by others was especially important following FEP diagnosis. The following excerpt exemplifies how one participant experienced disruption to their sense of belonging since their diagnosis and the need to establish new connections: “In order to feel healthy I need to create a new sense of belonging since I’ve become sick… I need to find a new tribe” (leaf). Connecting over shared experiences and similar interests were an important way that rangatahi whai ora fostered whanaungatanga. Some noted the value of knowing others who could relate to their experiences. The following excerpt reveals how shared experiences were not only connecting but validating: “Living with a flatmate who also experiences interesting realities makes it feel okay to be me” (written). However, rangatahi whai ora also described how finding connection over similar interests, and beyond the shared experience of psychosis, was important.

### Addressing Stigma

Rangatahi whai ora revealed how stigma had permeated their lives since they started experiencing FEP. They described “feeling judged” (written) by others and by society more generally. Sometimes, rangatahi whai ora experienced stigma from partners or family members (see *whanaungatanga*), or in healthcare settings. Other times, self-stigma was described, with participants feeling embarrassed or fearing others might ‘discover’ their psychosis. One participant described their experience of stigma on multiple levels as a nursing student:

…it was definitely a fear of stigma from the people that I was surrounded with in a healthcare environment… but also stigma from the partner that I was with at the time, the people I was surrounded [by]. I can remember screaming at the top of my lungs when I was in a fit ‘I don’t want to be schizophrenic’ and he was like ‘you’re not going to be schizophrenic’ (interview).

This stigma negatively affected the health of rangatahi whai ora by creating barriers to connecting with others. Participants also explained how stigma made it difficult to understand psychosis, including for themselves. One person explained: “Even I had a fear of stigma because I didn’t understand what was happening to me …I think stigma is a very confusing topic when you’re unwell and battling with it” (interview). This participant’s experience highlights how stigma can limit understanding of what psychosis is, which can delay acceptance of their diagnosis and moving forward. Close supporters were not immune to stigma either. A mother of one of the participants described the stigma she experienced from others:

Nobody understood you anymore and nobody really wanted to be with you anymore because people felt that if they associated with you, the shame and judgement… they would be coated with it…I’d say this is probably the biggest coat I’ve had in terms of judgement from society (whānau, interview).

On a societal level, stigma was pervasive in the everyday environment. Rangatahi whai ora described the media as being an important source. Similarly, the whānau member elaborated on the impact that stigmatising language in the media could have: “[The media] have a real role to play in the way they use the word psychotic… they really play on it and they have no idea what it means either” (whānau, interview).

Participants’ responses highlighted the harm of stigmatising language and offered ways that language could be shifted and reframed to address stigma. During group discussion, participants talked about how people with psychosis are often perceived as dangerous, yet they tend to be the ones who are most vulnerable. Some suggested using a strengths-based approach to describe those with FEP, such as “interesting,” “creative” or having an “active mind” as opposed to deficit framings like “psychotic” (leaf). Another participant related their psychosis to having a superpower: “I think about X-Men when we get together with our open minds, free speech, and independent thinking. The label ‘psychosis’ is dismissive of the experience” (group discussion).

The use of labels was discussed as having potential to be harmful and helpful; for instance, carrying the label ‘psychosis’ could be beneficial by connecting people with shared experiences (see *whanaungatanga*). However, the same label could be detrimental due to the associated stigma and inconsistency with one’s unique experience of psychosis. Rangatahi whai ora emphasised the importance of being able to choose their own labels: “If you give people a label it can be damaging or it can be helpful - people drop their personal expectations. People need to be able to choose, because labels can either empower or disempower and the goal should be empowerment” (group discussion).

Ultimately, participants dreamt of a world without stigma. They described wanting to openly talk about psychosis like other mental health topics such as anxiety and depression. As one participant noted, they desired to “live in a world where it is okay to be me, no matter how I am” (written).

### Finding Out Who I Am With Psychosis

Rangatahi whai ora described shifts in their identities due to FEP, such as feeling disconnected from themselves, a loss of identity, or feeling like a different person on medication. Hence, *finding out who I am with psychosis* emerged as essential to living well with psychosis in response to these shifts.

Making sense of their psychosis was as an important part of exploring their identity. Collectively, rangatahi whai ora revealed their psychosis potentially led to a deeper understanding of themselves. For example, one person recognised their psychosis as being a part of their identity crisis, which signalled the need to reconnect with themselves. The interpretations of what psychosis meant among rangatahi whai ora were highly personal and varied. One person interpreted their psychosis as a spiritual phenomenon, described as a ‘kundalini awakening’ (interview). Conversely, another person found that distancing themselves from spiritual explanations helped them to better understand their psychosis. The quote below reveals, not only did they gain a better understanding of their psychosis, but their psychosis enriched their life experience:

Having let go of that spiritual connotation allowed me to accept the kind of practical steps I needed to make… and [focus on] my routine. And that’s why it’s so important now, to actually feel well… It’s [psychosis] a bit scary at times, but it’s cool. It gives you a lot of perspective. And I don’t necessarily feel wiser, but I feel like I’m enriched by my experience now rather than scared of it (interview).

Understanding how psychosis intersected with the other facets of their identity was also important. For example, one participant explained how their neurodiversity and psychosis jointly shaped their identity and interpersonal connections. Similarly, discovering their own version of recovery informed their personal journey of living with psychosis. To some, recovery meant, “turning up every single day” (group discussion), while another described, “doing whatever [they] wished without [their] mood or state of mind getting in the way” (leaf). One reframed their recovery to living well *with* psychosis, rather than its absence:

I thought that recovery was going to be about living without psychosis. But now I aspire to have a life worth living in the absence of mental distress. I accept my experience. I can’t participate in the world the way others do. And that’s ok, so long as it is free of mental distress (group discussion).

As part of the process, self-acceptance was an important part of embracing who they were with FEP. One participant described the relief they experienced from accepting who they were with psychosis:

So, from a young age with that spiritual kind of ideology behind loss, you’re kind of looking for an outside remedy and searching for something that’s kind of what self-discovery and spirituality is, you’re searching for something. And when I had my psychosis, the searching for something became a frantic kind of pulling sensation to find out what was wrong. And then once I found out the diagnosis and accepted it…it was a huge relief to be like wow, that’s just my mind (interview).

Correspondingly, a lack of self-acceptance was a barrier to living well. Some participants noted the challenges of constantly putting themselves down (group discussion) or “comparing [themselves] to others” (written). This emphasis on self-acceptance and acceptance from others (see *addressing stigma*) reinforces the value of utilising positive and self-defined labels to signify acceptance.

Finding out who they were with psychosis also meant engaging in activities that felt meaningful. Some shared activities that helped them reconnect with themselves such as journaling, therapy, and listening to music. When asked about a potential intervention, participants explained that any activities should “fit with people’s interests” and “not necessarily [be] specified by the type of mental illness” (interview). Such responses highlight the importance of supporting rangatahi whai ora to follow their interests and aspirations defined beyond their psychosis. The goals and aspirations they shared pointed to the ambitions of their (re)discovered selves, whether they be making a difference by “helping others” (group discussion), wanting to “become a famous rapper and animator/actor” (leaf), “a games designer” (leaf), “embracing [their] inner child” (interview), or, more generally, “creating something original” and “learning new things” (leaf). One participant explained how following their interests was in fact an important way of discovering themselves on a deeper level: “You get healthy through exploring, through exploring what you are interested in, through exploring your desires because you find it works for you, through doing what you want to do. You learn through doing” (interview).

### Getting the Basics Right

A further factor that arose as important to living well with psychosis was *getting the basics right*. Rangatahi whai ora considered the ‘basics’ to be a wide range of routine behaviours that supported them to live well since their diagnosis. Getting the basics right involved (re)discovering and (re)adjusting daily behaviours and practices that improved their health and wellbeing, modifying behaviours that produced negative effects, and fine-tuning medication to balance the relief of distressing symptoms with minimising unwanted side-effects.

In part, getting the basics right meant learning about health-promoting behaviours, which ones were important to them, and being supported to engage with them: “Something I try to do to stay well is get the basics right – for me, this is sleeping, moving my body, connecting with friends, and doing activities I enjoy” (written). Through various creative platforms, rangatahi whai ora highlighted the importance of sleeping well (feeling rested, sleeping everyday), eating healthy (eating “proper” food, drinking water), physical activity (going to the gym, walking, swimming), relaxation (mindfulness, listening to music, video games), and doing activities that were important to them (e.g., table tennis, social interactions). Avoiding maladaptive behaviours was also stressed, such as spending too much time at home, watching porn, unhealthy eating, problematic use of social media, excessive partying, and substance use. Adhering to a good routine to help them engage with health promoting behaviours was underscored.

Having the right medication was also essential to living well. Getting the basics right meant that medication side effects had the least impact on their daily life as possible. Participants described the negative impacts that side effects had on their health including low mood, hand tremors, weight gain, acne, dry mouth, skin conditions and feeling like a “different person”.

### Collaborative Healthcare

The desire for collaborative healthcare emerged as a strong theme toward supporting good health and wellbeing among rangatahi whai ora. During a group discussion, they explained how the power dynamics and the ‘churn’ in the system - meaning the high turnover and rotations of staff - made it difficult to build trusting relationships with their healthcare providers. The interactions with therapists and consultants were described as “mechanical”, “transactional”, and “conditional [on progress]”, either frequently re-telling their story or not being asked in the first place such that important decisions were made on their behalf without context. These relationships were described as “paradoxical” because they were not conducive toward genuine recovery.

I don’t feel like I am being treated fairly. They don’t properly investigate. Every time I ask for a second opinion they say, ‘take the meds and see how you go in 6 months.’ My lack of trust with the doctors gets in the way of my recovery (written).

During such a confusing and vulnerable point in their lives, rangatahi whai ora desired relationships where they could work closely with their healthcare providers in a way they felt understood. They wanted to be asked questions, feel heard, respected, and have a say in important decisions about their health. For example, one person described a positive experience with EISP where they were not forced to take medication. Rangatahi whai ora emphasised approaches that focussed on their strengths, calling for “acceptance [of who they are]” and a focus on “progress, not perfection” (leaves), which required healthcare providers to take the time to know who they are and learn what is important to them: “Sometimes you want to talk without getting advice. They [therapists] focus on diagnosis, not caring for all the dimensions of your wellbeing. I am interested in shaman type relationships, trusted, people who just accept who I am and where I am at” (group discussion). However, few positive experiences of such approaches were provided.

Participants desired a collaborative approach in that it also involved key supporters. In some instances, rangatahi whai ora wanted an advocate to speak on their behalf. In other cases, whānau were seen as key people who could support them through the process. One participant described whānau members as being an important part of decision making around medication:

The doctors need to work closely with me and my family to make sure my medications have the least side effects possible to achieve my recovery. Because the side effects have made my mood be at an unliveable level to allow me to lead a healthy life (written).

### Understanding Psychosis

‘Understanding psychosis’ was critical to the health and wellbeing of rangatahi whai ora and emerged as a bi-directional exchange: 1) rangatahi whai ora seeking appropriate information to understand psychosis, and 2) needing others (wider society) to understand the lived experience of FEP.

Participants, including whānau, emphasised their need for relevant information about psychosis. Rangatahi whai ora sought information about psychosis at different stages (particularly FEP), medications and side effects, support for health behaviours (access to dietician, cooking classes, local activities) and recovery. They also wanted to know about diverse experiences of living with FEP, which they found challenging to access. Some encountered difficulty accessing appropriate information from services:

Something that was lacking in my EIS existence was education. I did not receive any education on psychosis early on which led to much confusion over what had and was happening to me. This “ignorance” led to large amounts of stress and worry as I couldn’t figure out what psychosis means to me in terms of its length, cause, and recovery. 90% of all of my education on psychosis was from my parents spending hours online to find information. The 10% from EIS only really started by at least the middle of the year (written).

Searching for information online also presented challenges. One person described the difficulty of finding appropriate information online specific to FEP, and people’s actual experience of it. One source, Talking Minds (https://www.talkingminds.co.nz/) was considered helpful and aided acceptance of their diagnosis.

I did find Talking Minds was really cool. It gives you a layout of medications. Like when you’re first starting out, and you’re like, ‘What the hell is this? I don’t have psychosis. I’m actually perfectly fine.’ And you’re in denial. It’s really good to be like, woah, okay, and you watch some of the videos and you’re just like, okay, yeah, maybe I am in psychosis (interview).

The quote above highlights the value of having access to appropriate information, but the barrier to accessing this type of information is concerning. Participants emphasised that information needed to be relevant to their individual needs given the diverse experiences of psychosis (see *finding out who I am with psychosis*) and available in a variety of formats, such as videos, programmes, talks, and written materials.

Rangatahi whai ora also indicated the need for others to understand what psychosis really is. They described how more lived experiences in mainstream media would help wider society understand the diverse experiences of psychosis and recovery toward addressing stigma (see *addressing stigma*). Moreover, they wanted to share their own experiences to help other rangatahi whai ora living with FEP.

### Access to Resources

Having access to appropriate resources and spaces was further critical to their health and wellbeing. In terms of spaces, some rangatahi whai ora highlighted the comfort and safety of their beds. Spending time in nature was also grounding. Some explicitly described the important relationship between their mental health and natural spaces, while others described immersing themselves in nature, such as going for beach walks, getting fresh air, or being amongst plants and flowers.

Having appropriate resources, such as financial security also played a critical role. Financial security was made evident through participants’ comments in the group discussion of “not having enough money” or a “suitable wage”. Time, information (see *understanding psychosis*), and appropriate healthcare (see *collaborative healthcare*) were all described as essential to having good health and wellbeing. As one whānau member highlighted, mental health wards were not safe for her son:

It’s [the acute mental health ward] not a safe place. And …there were two psychiatrists and they both agreed with us that it was not a safe place. And that’s why they were releasing him after a week. Each time he was in there, they said it’s not an appropriate place for somebody on the autism spectrum. They cannot provide for his needs (whānau, interview).

## Discussion

This study sought to explore how recent clients of EISP Wellington experienced good health and wellbeing in Aotearoa. The resulting themes outline seven aspects that are important for rangatahi whai ora to live well with FEP including *whanaungatanga*, *addressing stigma*, *finding out who I am with psychosis*, *getting the basics right*, *collaborative healthcare*, *understanding psychosis*, and *access to resources*. Many of these themes expand upon existing research. *Whanaungatanga* deepens previous findings on social and spiritual relationships (Chee et al., 2019; Hultsjö & Blomqvist, 2013; Jordan et al., 2020; Lal et al., 2014) to include connecting with nature. *Getting the basics right* illuminates that engaging in meaningful activities, relaxation, and finding the right balance of medication are not only important for rangatahi whai ora, but vital features of daily life. Physical activity, sleep, healthy eating, and mitigating recreational drug use (for some) are equally essential, similarly reported by Hultstjö and Blomqvist (2013). Our theme *access to resources* contributes appropriate healthcare, safe spaces, and access to nature as key resources in addition to prior findings of financial stability (Hultsjö & Blomqvist, 2013; Lal et al., 2014). *Understanding psychosis* reflects Chee and colleagues’ (2019) findings that young people with FEP seek early education on physical health and medication from health professionals. However, our theme further suggests that rangatahi whai ora want to learn about diverse experiences of FEP and recovery from others with similar experiences. They also wanted to help other young people by sharing their story. These findings illuminate how lived experience of FEP is critical for supporting rangatahi whai ora and wider society to understand psychosis.

The remaining themes were consistent with previous findings. *Addressing stigma* is comparable to Makdisi and colleagues’ (2013) findings on self-stigma, social stigma, and stigma by the health system. *Finding out who I am with psychosis* echoes results from similar studies that one’s identity must be reconstructed after diagnosis, for instance “rebuilding self” (Pitt et al., 2007), and ‘finding a new way to be me’ (Attard et al., 2017). Other research also notes how engaging in meaningful activities is critical to this process (Hultsjö & Blomqvist, 2013; Makdisi et al., 2013). The need for *collaborative healthcare* has been extensively discussed (Chee et al., 2019; Hultsjö & Blomqvist, 2013; Jordan et al., 2020; Makdisi et al., 2013; Pitt et al., 2007).

Collectively, the seven themes inform five processes for supporting rangatahi whai ora to live well with FEP in Aotearoa: *whakawhanaungatanga*, *reframing*, *creating space for young people*, *holistic approaches*, and *improving access to appropriate resources* (see Fig. 3; Table 1).

**Table 1:** Five processes that support rangatahi whai ora to live well with FEP

Process	Description	Underpinning themes
Whakawhanaungatanga	Supporting rangatahi whai ora to build meaningful relationships with their whānau, others with shared experiences and/or similar interests, health professionals, advocates, nature, and spiritual realm.	Whanaungatanga, Collaborative health care, Access to resources, Understanding psychosis
Reframing	Using a strengths-based approach to help rangatahi whai ora explore, describe, and share what living well with psychosis means to them and the diverse ways this is experienced.	Addressing stigma, Finding out who I am with psychosis, Understanding psychosis
Creating space for young people	Dedicating time and space for rangatahi whai ora to explore and enact what living well with psychosis means to them and how this translates into their everyday life. This involves asking rangatahi whai ora about their values and priorities and creating an environment to help them (re)discover their strengths and identities.	Whanaungatanga, Finding out who I am with psychosis, Getting the basics right, Collaborative healthcare, Understanding psychosis
Holistic approaches	Supporting rangatahi whai ora in a way that recognises, and tends to, the many dimensions of their health and wellbeing (e.g., physical, mental, whānau, spiritual) and how they are interrelated.	Whanaungatanga, Finding out who I am with psychosis, Getting the basics right, Understanding psychosis
Improving access to appropriate resources	Connecting rangatahi whai ora with existing resources (e.g., services, community, food, healthcare, etc.) that are relevant to their needs and addressing where there are gaps.	All themes

Note: This table presents the recommended processes to support rangatahi whai ora live well with FEP and the themes that underpin each process. While numerous links can be drawn between the themes and processes, the clearest links are presented based on what rangatahi whai ora have said within the descriptions of each theme

*Whakawhanaungatanga* is central to the wellbeing of young people living in Aotearoa (Carlson et al., 2022). Our findings point to numerous opportunities that support rangatahi whai ora through whakawhanaungatanga, building meaningful connections with their whānau, peers, service providers, spirituality, nature, and more. *Reframing* also arises as a key tactic to help address the stigma rangatahi whai ora face and to (re)discover their identity with psychosis. Our participants favoured ‘strengths-based’ language that highlights the strengths of their psychosis rather than the deficits, and an emphasis on the similarities they share with others. Likewise, Jordan et al. (2020) suggest ‘positive reframing’ helps rangatahi whai ora create new meaning and knowledge toward change and recovery, for instance by framing psychosis as being useful, a catalyst for positive change, and a therapeutic or spiritual phenomenon. Using positive language and labels to reframe psychosis, particularly among healthcare providers and wider society, presents a significant opportunity to address the barriers that stigma poses for rangatahi whai ora by promoting inclusion and whakawhanaungatanga in occupational and healthcare settings, as well as fostering self-acceptance.

Our findings also reveal that health and wellbeing among rangatahi whai ora is clearly holistic, which requires an equally *holistic approach* in response. Our participants felt safe and supported by connecting with people, the spiritual realm, and the natural world. Further, ‘getting the basics right’ entailed a wide range of factors integral to their daily lives that impacted their mental, physical, spiritual, and social wellbeing. The multidimensional nature of wellbeing among this population has been similarly reported, encompassing psychological, physical, emotional, moral, financial, spiritual, and social aspects (Lal et al., 2014). Young people have voiced their desire to learn how these aspects integrate (e.g., between mental and physical health) earlier in their FEP journey (Chee et al., 2019), which calls for more holistic health support.

Enacting whakawhanaungatanga, reframing, and a holistic approach to support rangatahi whai ora requires a significant shift in existing systems and processes to *create space for young people*. Whakawhanaungatanga demands space and stability to form meaningful relationships and connection. Identifying appropriate frames is a personal journey that requires listening to, and working *with*, rangatahi whai ora to (re)discover their identities and find appropriate language that supports their unique experiences of FEP and recovery. A holistic approach also entails creating space for them to discover behaviours that enhance their health and wellbeing and how these translate into daily life. In addition to creating space, *improving access to appropriate resources* is imperative to meet the diverse needs of rangatahi whai ora based on their identities, interests, social environments, and contextual constraints. For example, Grattan et al. (2024) report that Māori face more systemic barriers, yet comparable rates of psychosis-related distress compared to non-Māori. They suggest that Māori experiences of FEP are highly nuanced and that cultural factors may be protective when interpreting psychotic experiences in some cases thus highlighting the need for culturally appropriate care.

This study showcases the use of creative platforms to support rangatahi whai ora to share their stories. The importance of using arts-based and creative qualitative methods in healthcare research is well-recognised with photovoice, participant-elicited drawings, poetry, and storytelling as valid ways of collecting phenomenographic data (Boydell et al., 2012). Participant-elicited drawings and photographs have previously been used to investigate how people experience psychosis (Attard et al., 2017; Lal et al., 2014). This research builds upon these studies by enabling participants to express themselves using a qualitative platform of their choice, including written and verbal responses to prompts, group discussion, and artistic outlets such as drawing, painting, and sculptures. Some types of data required greater interpretation from researchers than others. For example, brief captions on drawings offered fewer insights compared to the responses shared through interviews, where further explanation could be prompted. However, several participants were deterred by speaking in group discussions or interviews and preferred expressing themselves creatively. Thus, this variety helped further reduce the barriers to self-expression and enabled data to be captured that may not have in other formats.

This study offers several notable strengths. First, our findings contribute seven themes that collectively extend our understanding of what young people with FEP deem necessary for improving their health and wellbeing as well as five key processes to enact appropriate support. This project also provided a platform for rangatahi whai ora to share a relevant vision of what it means to live well with FEP. Our research further highlights the added value of working alongside those with lived experience of FEP. Our lived experience researchers guided the research process, which strengthened our methods and insights at each stage. In addition, both participant and researcher experts by lived experience, co-designed the workshop (see Jenkins et al. 2023), which outlines a new and empowering way of conducting research. The resulting workshop enabled participants to share their stories by their choice across a wide variety of platforms including a mix of creative and art-based methods. This approach enriched the data with stories that may have been lost if constrained to a single platform. Finally, our findings add to a small pool of evidence relevant to rangatahi whai ora living in Aotearoa. For example, the emphasis on connecting with nature may speak to the cultural values held by those living in this context.

Several limitations should be considered when interpreting the study findings. While the ability to inform practice in the local context is a strength, the generalisability to other contexts, even other localities within Aotearoa, is limited. Additionally, this research focussed on rangatahi whai ora in the early stages of psychosis thus the findings may be less applicable to adults who have had multiple episodes. However, the holistic nature of findings and consistency with other research suggests their relevance. The data were collected at a single time point, representing only a cross-section of experiences among rangatahi whai ora. Yet, health and wellbeing with FEP is a dynamic and everchanging phenomenon. Future research could investigate cohorts at later stages of their psychosis or collect data at multiple timepoints to enrich the results reported here. Lastly, the data only represents participants that were engaging with EISP. Future research is needed to explore the experiences of those with even greater barriers to accessing services, which could expand upon the aspects reported in this study, such as deeper insights regarding access to resources.

Supporting rangatahi whai ora to live well with FEP requires a collaborative and comprehensive effort between health and social service providers, rangatahi whai ora, their whānau, community groups, experts by lived experience, researchers, and other key stakeholders. We hope the seven themes and five processes reported here can guide future support development within the local region with the potential to be adapted more broadly. We also hope our methods guide and inspire future work with lived experience experts in a genuinely collaborative and creative way that enhances the research process and beyond.

## Footnotes

1 “Rangatahi whai ora” refers to young people in search of wellbeing. In this study, rangatahi whai ora is used to denote participants who were recent clients of EISP.

2 “Whānau” is commonly translated to family but can also refer to close relations that are non-kin, such as close friends and other key supporters. This broader interpretation of whānau was used to refer to participants’ key support persons.

3 Whakawhanaungatanga is a holistic and relational concept originating from matauranga Māori which encompasses the building of relationships between people, the spirit world, and the natural environment.
